# Positive relationship between seasonal Indo-Pacific Ocean wave power and SST

**DOI:** 10.1038/s41598-021-97047-3

**Published:** 2021-08-31

**Authors:** Sukhwinder Kaur, Prashant Kumar, Evan Weller, Ian R. Young

**Affiliations:** 1 Department of Applied Sciences, National Institute of Technology Delhi, Delhi, India; 2 School of Environment, University of Auckland, Auckland, New Zealand; 3 Department of Infrastructure Engineering, University of Melbourne, Melbourne, Australia

**Keywords:** Climate sciences, Atmospheric science, Climate change, Ocean sciences, Physical oceanography

## Abstract

The influence of increasing sea surface temperatures (SSTs), in response to greenhouse warming, on wave power (WP) remains uncertain. Here, seasonal relationships between SST anomalies and mean and extreme WP over the Indo-Pacific Ocean are examined. Overall, seasonal WP has significantly increased over much of the Pacific, Indian, and Southern Ocean by 1.21–3.10 kW/m dec^−1^ over 1979–2019. Contributions from wave characteristics, namely significant wave height (SWH) and peak wave period (PWP), to changes in WP show that SWH contributes most in extra-tropical regions, and PWP most in tropical regions. Further, seasonal relationships between SST anomalies and WP indicate that increases in WP are also seen during strong El Niño years in December–February, and in-phase combinations of El Niño and positive Indian Ocean Dipole (IOD) events during June–August and September–November. Results highlight both long-term increasing SSTs and climate variability roles for inducing large-scale seasonal WP changes throughout the Indo-Pacific.

## Introduction

Climate change, whether driven by internal natural variability or anthropogenic forcing, can prompt a shift in the occurrence or strength of extreme weather and climatic events. Most notably is the significant impact caused by the rise in greenhouse gases (GHGs) due to human activities. As a result, increases in oceanic temperatures have had a diverse effect on ocean–atmosphere circulation, which in turn have significant impacts on pressure gradients, winds, and wave height or power^1^. Wave power (WP) helps to describe changes in wave climate, and is also a potential and powerful resource for clean renewable energy as society looks for alternatives to fossil fuels^1,2^. Therefore, a comprehensive understanding of the relationship between the oceanic warming (commonly depicted by sea surface temperature (SST) increase) and WP is required at different spatial and temporal scales.

Numerous studies have previously reported observed trends in the seasonal and annual wind-wave climates at the global^3–5^ and regional scale, particularly in the North Atlantic (NA)^6,7^, North Pacific (NP)^8,9^, Northern Hemisphere^10–12^, and Southern Hemisphere^13,14^. Overall, increasing trends are predominantly found in extreme significant wave height (SWH) over the northeast NA in winter (January–March) and central NP in winter and spring (April–June). Larger increases in SWH were observed year-round in the Southern Ocean, although largest during the Southern Hemisphere winter (July–August)^13,15,16^. Further, studies suggest that anthropogenic forcing is a key driver of such changes in wave heights^12,13,17^. Subsequently, other wave climate indicators, such as wave period and/or direction, have been utilized to identify the underlying properties in the changes to mean and extreme SWH^18–21^. Inherently, being a measure of the energy transported by ocean surface waves, transmitted over cumulative periods of time from the atmosphere to the surface motion of the ocean^22^, WP consists of vital information about SWH and peak wave period (PWP) (see methods, Sect. 2.2). Hence, WP is a relevant climate indicator for examining long-term change in global and regional wave climate conditions^1^.

Early studies provided an overview of the distribution of global coastal WP density based on limited data as measured by marine ships and wave rider buoys^23,24^. Owing to the rapid development of marine remote-sensing and numerical simulation technologies, increasing amounts of hindcast wave and satellite altimeter data have been used to analyze wave energy resources^25–27^. For example, Cornett^26^ proposed unique monthly and seasonal variability indices to quantify variations in WP. Global information on the distribution and variations in mean WP have been examined utilizing models such as Wave Watch-III (WW3)^25,27–29^system and ECMWF WAM^30^ but only for periods less than 10 years. More recently, the long-term seasonal and interannual variations in global mean WP were reported by Reguero et al. ^31^ using WW3 model data over a 61-year period (1948–2008) and by Rusu & Rusu^32^ based on ERA5 data for a 30-year period (1989–2018). Martinez & Iglesias^33^ provides global details of the Wave Exploitability Index along with the mean WP using ERA5 data for the period 1979–2019. Several regional studies including the NA^34–36^, NP^37–39^, Black Sea^40^, Australian coasts^41,42^, and shelf seas of India^43–46^ have also been carried out.

Perturbations in SST can induce fundamental changes in the large-scale general circulation that subsequently have an impact on the wave climate through responses in the surface winds^47–50^. On an interannual time scale, natural modes of climate variability drive changes in the wind and wave climates via perturbations in SSTs unique to each mode. However, over the tropical Indo-Pacific region, changes in SST in response to global warming have been shown to be important for tropical cyclone intensity and precipitation^51^. Indeed, several studies have examined changes in SST as a climate change indicator in the historical wave climate^51–53^. Recently, Reguero et al.^1^ investigated the WP relationship with various climate variability modes, such as El Niño–Southern Oscillation (ENSO) and the Atlantic Multidecadal Oscillation (AMO), and demonstrated that recent increases in global WP were associated, both spatially and temporally (seasonal), with oceanic warming driven by natural climate variability modes. More recently, Odériz^54^ analyzed the global seasonal (particularly December–February (DJF) and June–August (JJA)) WP and its relationship with oceanic warming based on three different wave climate types. Overall, large-scale natural climate variability can enhance or dampen the consequences associated with global warming^55^. Therefore, a better comprehension of the seasonal relationship between SST increases (driven by either natural climate variability or anthropogenic forcing) and WP, particularly extremes, in each ocean basin requires more attention. Here, seasonal interrelation between oceanic warming (SST increase) and WP is analyzed using reanalysis data for a 41-year period (1979–2019) with a focus on the Indo–Pacific Ocean. Long-term trends in time series of the seasonal mean WP and SST averaged over oceanic regions are examined. A stationary generalized extreme value (GEV) analysis is applied to focus on the extremes in WP, SWH, and PWP, and together with the climatology of WP, including the individual contribution of SWH and PWP, seasonal interrelations between the WP and ocean warming (SST increases) are explored in detail. In addition, various temporal correlations between the WP and SST anomalies are also breifly examined for further insight into the relationship between WP and SST anomalies.

## Data and methods

### Data

The fifth generation of the European Centre for Medium-Range Weather Forecasts (ECMWF) reanalysis product, referred to as ERA5^56^, with a temporal resolution of six hours (constructed from the two-dimensional wave spectra at an hourly temporal resolution), was used for analyzing mean and extreme WP over the 41-year period from 1979–2019 in the Indo–Pacific region for the four seasons (i.e., December–February (DJF, winter), March–May (MAM, spring), June–August (JJA, summer), and September–November (SON, autumn). To calculate WP, seasonal mean and extreme SWH (combined wind sea and swell) and PWP are derived using ERA5. In addition, seasonal SST data was also obtained from the ERA5 archive. As the latest ECMWF reanalysis product, ERA5 has higher spatial and temporal resolution compared to ERA-Interim^57^ and presents several improvements, such as improved representation of the troposphere, tropical cyclones, and precipitation cycle. All ERA5 data used here were downloaded from the ECMWF site (https://www.ecmwf.int/en/forecasts/datasets/reanalysis-datasets/era5/), either with a horizontal resolution of 0.5° × 0.5° (i.e. SWH and PWP) or 0.25° × 0.25° (i.e., SST).

### Methods

In essence, WP measures the transmission of energy by/through air-sea exchanges and used for wave motion^22^. For irregular waves, the WP is derived from wave spectral parameters^26,39^ as:

1 $$WP=\frac{\rho {g}^{2}}{4\pi }\left(\frac{{T}_{e}{H}_{s}^{2}}{16}\right),$$

where *g* is the acceleration due to gravity, ρ is the sea water mass density (~ 1028 kg/m^3^), ${H}_{s}$ is the SWH, and T_e_ is the energy period. Wave energy period was obtained using the approximation ${T}_{e}=\alpha {T}_{\mathrm{P}}$, where T_p_ denotes the PWP and the parameter $\alpha =1$ was used accordingly^58^. To describe extremes in wave parameters, the seasonal maxima at each defined grid point are fit to a stationary GEV distribution as in previous studies^59–64^. The cumulative distribution function of GEV distribution is given as:

2 $$F(x,\mu ,\sigma ,\xi ) = \left\{ {\begin{array}{*{20}l} {\exp \left[ { - \exp \left( { - \frac{x - \mu }{\sigma }} \right)} \right]}, \; \xi = 0 \\ {\exp \left[ { - \left( {1 + \xi \frac{x - \mu }{\sigma }} \right)^{{ - \xi^{ - 1} }} ,\xi \ne 0,1 + \xi \frac{x - \mu }{\sigma } > 0} \right]} \\ \end{array} } \right.,$$

where $-\infty <\mu <\infty , \sigma >0, and-\infty <\xi <\infty$ represent the location, scale, and shape parameters, respectively.

Next, mean seasonal contributions of the SWH term (Hs^2^) and PWP term (Tp) in Eq. 1 to the WP over the Indo-Pacific were obtained through a ratio and proportion method. For example, to calculate the contribution of SWH and PWP to the WP at any given location, firstly the ratio of Hs^2^ and Tp individual contributions in WP is computed (i.e., a: b ratio whereby a and b represent Hs^2^ and Tp, respectively). The contribution of SWH and PWP to WP are estimated as [(a/(a + b))*100%] and [(b/(a + b))*100%], respectively.

Once the Hs^2^ and Tp individual contributions in WP are obtained, time series of the seasonal mean WP is computed by averaging over the global (90° S–90° N, 0° E–360°), Southern (80° S–40° S, 0° E–360°), Indian (90° S–30° N, 20° E–120° E), Pacific (90° S–90° N, 120° E–70° W), and Indo-Pacific (90° S–90° N, 20° E–70° W) ocean basins.

Long-term trends were calculated using linear regression analysis. The statistical significance of the trends was calculated using the Mann Kendall (MK) test^65^. The standard P-values obtained from the MK test are based on the assumption of independence between the observations. Given this, it’s important to check the autocorrelation in a given series and, if necessary, adjust the MK test. To avoid autocorrelation in the time given series, we followed the Wang and Swail^11^ approach. The WP and SST were correlated with each other at various temporal scales, to quantify their interconnection, the Pearson’s correlation coefficient is used. The significant correlation at the 99% and 95% level of confidence is computed by using a two-tailed Student’s t test.

## Results

### Seasonal wave power

**Figure 1:**
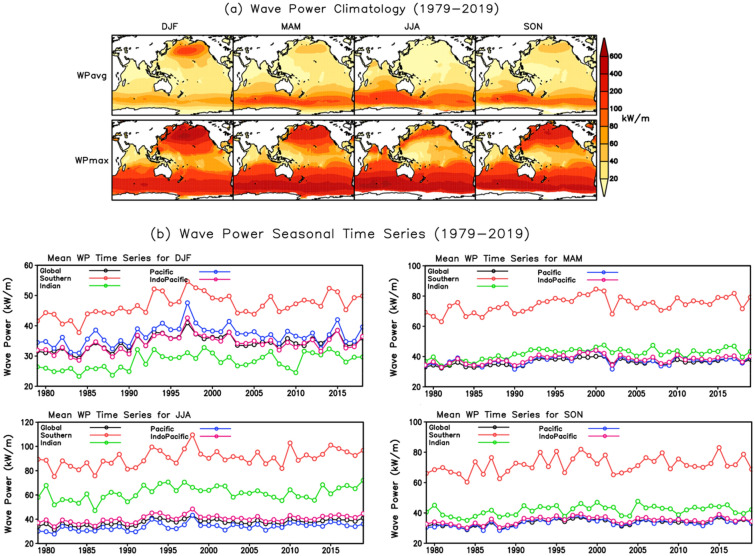
(**a**) Climatology patterns of the seasonal mean and extreme WP in the Indo-Pacific Ocean for the period 1979–2019. The unit of WP is kilowatts per meter (kW/m). (**b**) Seasonal mean WP time series for DJF, MAM, JJA, and SON, averaged over the global, Southern(80° S–40° S, 0° E–360°), Indian (90° S–30° N, 20° E–120° E), Pacific (90° S–90° N, 120° E–70° W), and Indo-Pacific (90° S–90° N, 20° E–70° W) oceans.

The climatological patterns of the ERA5 seasonal mean and extreme WP (WPavg and WPmax) for the 41-year period (1979–2019) over the Indo–Pacific Ocean are displayed in Fig. 1a. Similar maps of seasonal mean and extreme SWH (Havg and Hmax) and PWP (Pavg and Pmax) are provided in Supplementary Fig. S1. Here, the mean climatology was acquired by taking the average over the 41-year period, and the extreme climatology is represented by the location parameter of the GEV distribution. High spatial correlations, ranging from 0.95–0.97, depict the close association between the seasonal mean and extreme WP, whereby spatial patterns show similar features and variations. Further, the seasonal WPavg and WPmax climatological patterns bear resemblance to the Havg and Hmax climatological patterns (*c.f.*Fig. 1a and Supplementary Fig. S1a). Highly significant correlations (0.96–0.97) between seasonal mean WP and SWH occur during all seasons, with spatial correlations between the seasonal extreme WP and SWH even higher (0.97–0.98). This indicates that both mean and extreme WP have a strong association with SWH^37–39^.

Large seasonal variation in WPmax occurs over the extra-tropical and subtropical regions (Fig. 1a lower panel). In the Northern Hemisphere (NH), during DJF, the Icelandic Low and Aleutian Low produce higher pressure gradients along the well-defined wind belts, and stronger surface winds, consequently enhancing the extra-tropical WPmax^54,66^. In JJA, the northern basins are dominated by the Icelandic and Pacific High anticyclones, resulting in stronger subtropical and weaker extra-tropical winds and wave climate^54^. In the Southern Hemisphere, the high and low-pressure belt is rather constant and westerly winds and waves are present throughout the year, with higher energetic conditions in JJA^31,54^.

To understand the individual roles SWH and PWP play in enhancing or reducing WP throughout the year, mean seasonal contributions of the SWH term (Hs^2^) and PWP term (Tp) to the WP over the Indo-Pacific were examined (Supplementary Table 1 and Supplementary Fig. S2). Overall, SWH predominantly contributes to WP in the extra-tropical regions of both hemispheres (about 65–80%), except for the NP during JJA, where SWH and PWP contribute to WP somewhat equally. Conversely, PWP largely contributes to WP in the tropical latitudes and regions such as the AS and BOB (about 54–82%). Therefore, SWH dominates WP in the most active wave generation zones where wind-seas are frequently observed, whereas PWP dominates WP where swells govern the wave climate or SWH is small (Supplementary Fig. S3).

Time series of the seasonal mean WP averaged over different ocean basins (i.e., the global, Southern, Indian, Pacific, and Indo-Pacific) for 1979–2019 are also presented in Fig. 1b. Overall, strong seasonality in WP is evident across the different oceans, yet WP is largest in the SO year-round. Similar seasonality has been reported in SO wave height, period, and direction^15,19,20,67^. The WP over the Indian Ocean (IO), which is dominated by swells from the SO year-round^68,69^and also experiences monsoons over the tropics in JJA^61,70,71^, is larger than the WP averaged globally and in the Pacific Ocean (PO) during MAM, JJA and SON (Fig. 1b). In DJF, the WP over the PO is larger than the global and IO averages (Fig. 1b).

### Seasonal trends in wave power and SST

The increases in WP and SST per decade (dec^−1^) are calculated for the same regions and provided in Table 1. Overall, WP and SST has increased globally and in all regions of the Indo-Pacific over the period 1979–2019 yet increases are shown to be significant only for certain seasons and regions (Table 1). Globally averaged seasonal WP has significantly increased in JJA by 1.08 kW/m dec^−1^, as do SSTs across the same region and season (0.13 °C dec^−1^). The largest increase in WP is found over the SO in JJA, with a significant increase of 3.10 kW/m dec^−1^, which is in agreement with other authors^13,31,32,54^. Further, WP over the PO has also significantly increased in JJA, by 1.21 kW/m dec^−1^. As in JJA, the low and high-pressure belts induce the subtropical and sub-polar winds, which intensify the wave power over the PO^54^. In addition, PO in JJA has experienced the highest warming in SST of 0.14 °C dec^−1^. Similarly, WP and SST exhibit a significant upward trend across the IO in DJF (1.29 kW/m dec^−1^ and 0.13 °C dec^−1^) and MAM (1.58 kW/m dec^−1^ and 0.11 °C dec^−1^). Lastly, WP has also significantly increased in the SO in MAM and SON, by 2.55 kW/m dec^−1^ and 1.50 kW/m dec^−1^, respectively, supporting an intensification of the wave climate in SO, as reported by other authors^31,32,54^. The SO has experienced warming in SST of 0.04 °C dec^−1^ in SON. Interestingly, WP has not significantly increased in Indo-Pacific Ocean year-round, and this region experiences the warming of 0.09–0.14 °C dec^−1^ throughout the year. Here it is also found that the PO and GO in DJF, MAM, and SON, as well as the IO in JJA and SON, shows a non-significant increase in WP, consistent with previous studies^72–74^, and these regions experienced significant increases in SST between 0.09–0.14 °C dec^−1^.

**Table 1:** Linear trends in the seasonal WP and SST over the period 1979–2019.

	DJF	MAM	JJA	SON
**WP increase (kW/m dec**^**−1**^**)**				
Global ocean	0.89	0.94	1.08*	0.61
Southern ocean	1.40	2.55*	3.10*	1.50*
Indian ocean	1.29**	1.58**	1.50	1.14*
Pacific ocean	0.75	0.56	1.21**	0.54
Indo-Pacific ocean	0.83	0.88	1.16	0.51
**SST increase (°C dec**^**−1**^**)**				
Global ocean	0.11**	0.10**	0.13**	0.14**
Southern ocean	0.03	− 0.01	0.04	0.04*
Indian ocean	0.13**	0.11**	0.11**	0.13**
Pacific ocean	0.10**	0.09**	0.14**	0.14**
Indo-Pacific ocean	0.10**	0.10**	0.13**	0.14**

Statistically significant increases at the 95% and 99% level of confidence are marked with * and **, respectively.

### Seasonal relationships between wave power and SST over indo-pacific ocean

**Figure 2:**
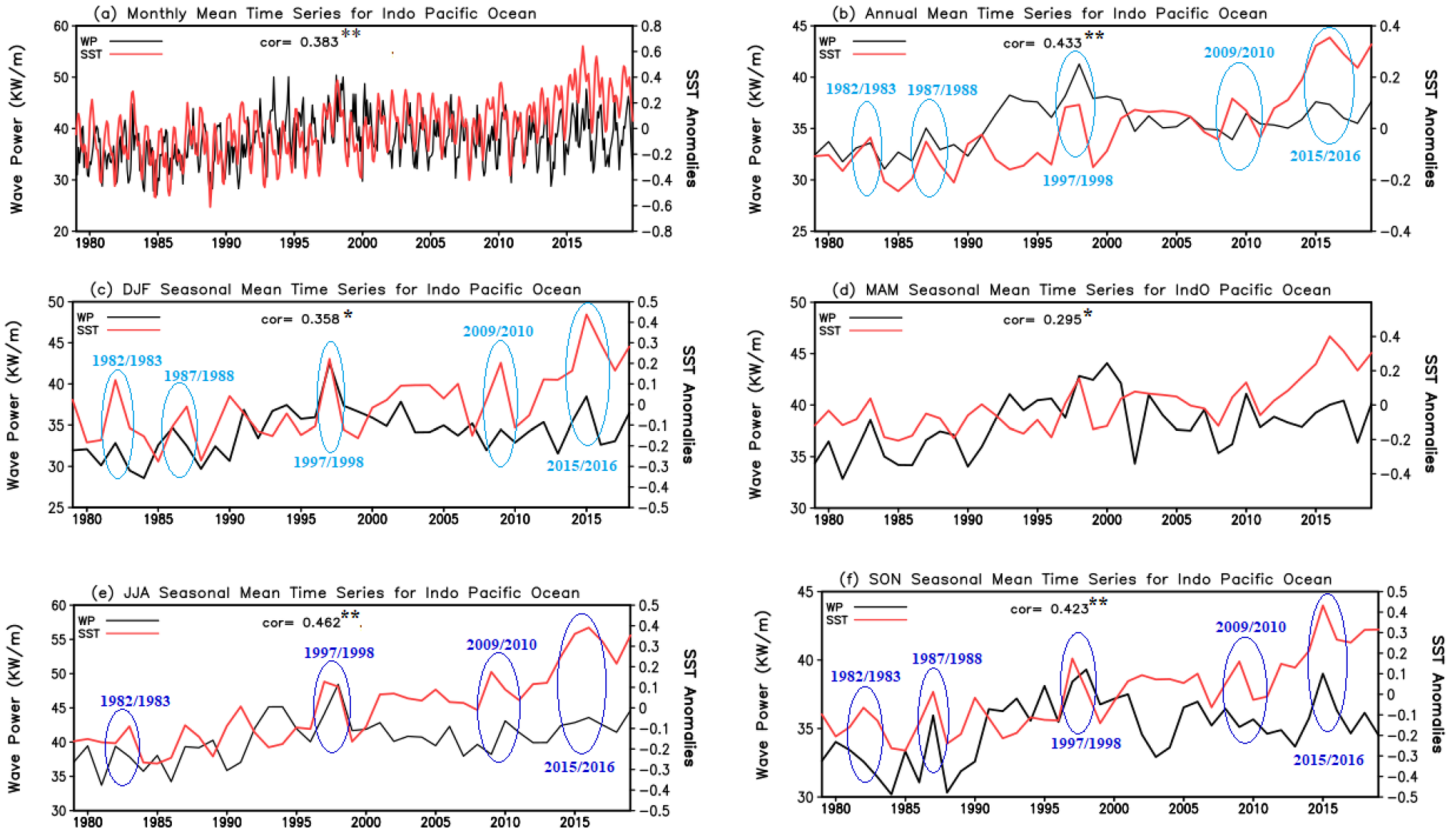
(**a**) Monthly, (**b**) annual, (**c**) DJF, (**d**) MAM, (**e**) JJA, and (f) SON mean time series of the WP (black, left vertical axis) and SST anomalies (red, right vertical axis) over the Indo-Pacific Ocean for the period1979–2019. Statistically significant correlations at the 99% and 95% level of confidence are indicated by ** and *, respectively, in each panel. Years with strong El Niño and/or positive IOD(pIOD) events are highlighted with blue circles, where light and dark blue, respectively, indicate El Niño only in DJF, and El Niño with pIOD in JJA and SON.

Figure 2 provides time series of WP and SST averaged over the Indo-Pacific Ocean (90° S–90° N, 20° E–70° W) at various temporal scales: monthly (Fig. 2a), annually (Fig. 2b), and seasonally (Fig. 2c–f) for the period 1979–2019. Significant correlations between WP and SST anomalies are seen at both the monthly (0.383, p-value < 0.01) and annual (0.433, p-value < 0.01) time scales, suggesting an association of SST anomalies with WP in the region. In addition, the relationship between SST anomalies and WP is found to be strongest during JJA and SON, with significant correlations (at the 99% level of confidence) of 0.462 and 0.423, respectively.

In the Indo-Pacific region, major modes of interannual and decadal natural climate variability (i.e., ENSO, IOD, and PDO) are dominant factors for inducing periods of strong oceanic warming that may cause an increase in WP. Figure 2 also highlights the strong El Niño events, as well as in-phase combinations of positive Indian Ocean Dipole (pIOD) and El Niño events (annotations overlaid on Fig. 2). Indeed, significant increases in WP are found during the strongest El Niño years (light blue circles in Fig. 2b–f) and years with in-phase combinations of pIOD and El Niño during JJA and SON (dark blue circles in Fig. 2e,f). A relatively weak relationship between SST anomalies and WP is found during MAM, coinciding with when ENSO and IOD are weak or not active. This suggests that there is a significant connection between WP and dominant interannual climate variability modes such as ENSO and the IOD in the Indo–Pacific. At longer time scales, WP and SST anomalies exhibit slow cycles somewhat consistent with PDO phase transitions. For example, the PDO underwent a positive–negative-positive phase transition during the period 1979–2019. The WP during the negative phase over the 2000s (often referred to as the global warming hiatus, Watanabe et al.^75^) is decreased compared to the 1990s, and appears to increase again after about 2014–2015, when the PDO shifted more positive. Therefore, it is important to further quantify the influence of natural climate variability on the mean and extreme WP over the wider Indo–Pacific Ocean region, yet out of scope of the present study.

**Figure 3:**
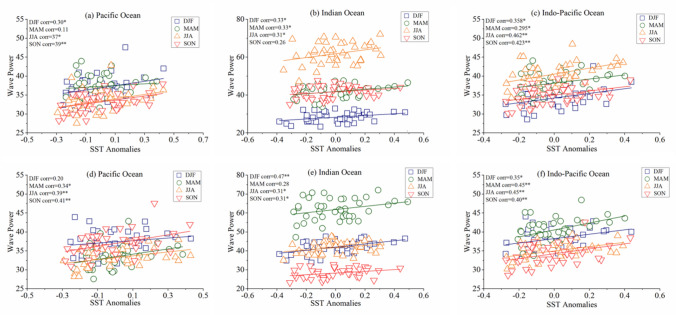
Seasonal interrelation between the mean WP and SST anomalies over the (**a**) Pacific Ocean, (**b**) Indian Ocean, and (**c**) Indo-Pacific Ocean over the period 1979–2019. Statistically significant correlations at the 99% and 95% level of confidence are indicated by ** and *, respectively. (**d**–**f**) The same as in (**a**–**c**) except between SST during a given season and WP one season after (i.e. time lag = 1).

Scatter plots between the seasonal mean WP and SST anomalies with no time lag and lagged by one season, averaged over the Pacific, Indian, and Indo-Pacific Oceans for the 41-year period from 1979–2019 are displayed in Fig. 3. In DJF, the El Niño events enhance the extra-tropical wave climate in the Northern Pacific as the ENSO positive phase is connected with a stronger/strengthened Aleutian Low^76^, which further intensified when ENSO and PDO both are in a positive phase combination, resulting in a strong correlation between SST warming and WP (0.30, p-value < 0.05) over PO (Fig. 3a). These findings are consistent with the previous studies that have examined the impact on wave parameters^62,77^. Further, this correlation over PO enhances in SON (0.39, p-value < 0.01) and JJA (0.37, p-value < 0.05), as in JJA, tropical cyclone activities over the PO (particularly in western North Pacific (WNP)) are amplified by El Niño events^39,54^. Whereas in SON (the El Niño development year), the deepening of East Asian trough, as well as the intensification and more frequent northward shift of the storm tracks across WNP, increases the wave climate over PO^77,78^. Likewise, in IO, La Niña (a pattern that is reversed during El Niño) increases the wave climate during DJF^62,68,77^, due to an increase in pressure gradient between the IO and eastern Pacific^62,77^, and consequently SST strongly correlates with WP (Fig. 3b,e). While, El Niño reinforces the wave climate over the IO during MAM^68,77^, resulting strong association between SST warming and WP (0.33, p-value < 0.05) (Fig. 3b). In JJA and SON, the La Niña events enhances the wave climate in the IO^54,61,68^, which amplified further when La Niña coincides with negative IOD events^61^, as well as negative PDO events^54,79^, and hence WP and SST correlates with each other in JJA (0.31, p-value < 0.05) and SON (0.31, p-value < 0.05) (Fig. 3b,e). Further, the wave climate in the SO which have been previously analyzed as significantly affected by Southern Annular Mode (SAM) with positive SAM impact at high latitudes and negative at mild-latitude year-round, which varies meridionally across seasons^13,61,77^, and also influences (weaker influence) the tropical and extra-tropical wave climate^54,61^. Overall, the warming in the Indo-Pacific Ocean (i.e. ENSO, IOD, SAM, and PDO) correlates with increases in the WP, resulting significant positive correlation between WP and SST throughout the year (Fig. 3c,f), ranging between 0.295–0.462. This indicates that seasonal variations in WP over both the IO and PO are intricately linked with SST warming, either in the same season or with a slight lag in the response.

**Figure 4:**
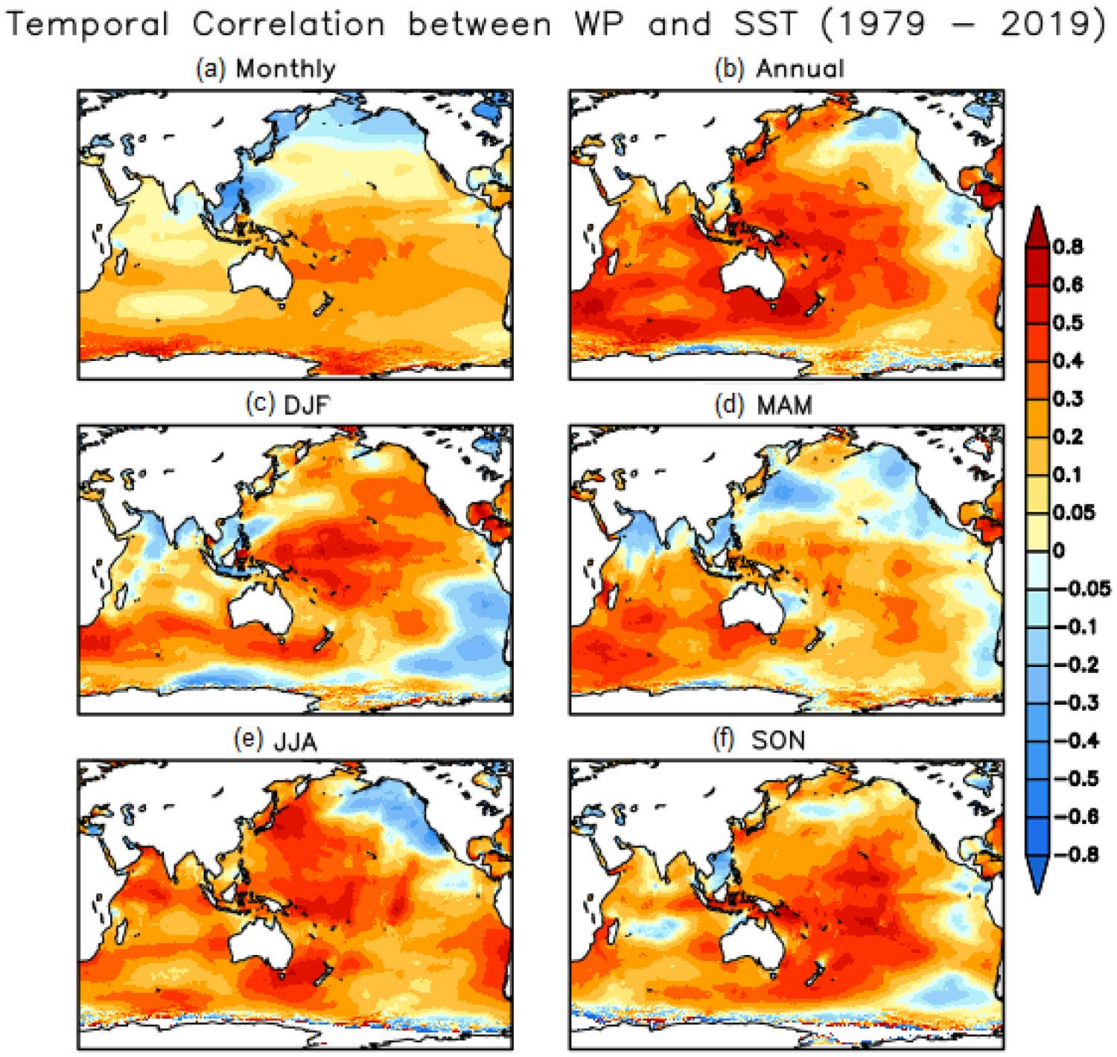
Temporal correlation patternsbetween the mean WP and SST for (**a**) monthly, (**b**) annual, and seasonal (**c**) DJF, (**d**) MAM, (**e**) JJA, and (f) SON time scales in the Indo–Pacific Oceanover the period 1979–2019.

Similarly, temporial correlations patterns between the WP and SST anomalies for the 41-year period reveal positive correlations ranging from 0.2–0.5 at monthly scale over the majority of the Indo-Pacific, yet negativecorrelations in the South China and Phillipine (SCP) Seas, BOB, and NP (Fig. 4a). Correlations increase (up to ~ 0.8) at the annual scale, except in theeastern Pacific and near Antarctica (Fig. 4b). In DJF,significantly high correlations (~ 0.5–0.8) exist over the equatorial PO and SIO (extending south of Australia), are more widespread in JJA and SON, yet weak in MAM, consistent with weak correlations overall in the average WP and SST anomalies (Fig. 4).

## Summary and conclusions

This study investigated the interrelation between ocean warming (SST increases) and WP in the Indo-Pacific Ocean using ERA5 reanalysis data over the period 1979–2019 at different temporal and spatial scales. Firstly, seasonal variations in the climatological patterns of the seasonal mean and extreme WP, SWH, and PWP reveal that large WPmax is evident in SO year-round, and strongest (lowest) in JJA (DJF), related primarily to an increase in SWH presumably from increased wind energy^21,25^. Similarly, increases in WPmax also occur over the NIO during JJA, and NP in DJF. Overall, SWH contributes most to the WP (up to ~ 80%) in the extra-tropical regions of both hemispheres and PWP contributes most to WP in the tropical latitudes (up to 82%). Although the SWH seasonal variations are similar to the WP seasonal variations, they are intricately different. For example, WP provides information about the energy carried from the ocean waves with differing amplitudes, whilst SWH provides information about the amplitude of the waves. An extreme wave contains high ocean wave energy or WP and a low amplitude wave contains low energy or WP. Therefore, WP provides a measure of the energy generated through ocean waves. Such wave energy can be transferred into renewable energy by using various energy convertors along the coastline, and consequently, WP is an important climate indicator providing such information about the available renewable energy resources and its variability.

Next, time series of the seasonal mean WP averaged over the different regions of the Indo-Pacific Ocean were examined and shown to exhibit significant increases in WP of varying degree across seasons. Overall, large significant increases in WP have occurred over the PO (1.21 kW/mdec^−1^) in JJA, the IO (1.29 and 1.58 kW/mdec^−1^) in DJF and MAM, and the SO (ranging 1.50–3.10 kW/mdec^−1^) in all seasons except DJF. Seasonal increases in WP somewhat coincide with the largest long-term seasonal increases in SST, except in the SO where no significant change is evident in SSTs. The seasonal interrelation between WP and ocean warming (SST increases) was further investigated through the relationship between seasonal WP and SST anomalies during the same season, and the WP one seasonal after the SST anomalies. From year-to-year, the seasonal mean WP and SST anomalies over the Indo-Pacific Ocean exhibit significant relationships whereby increases in WP are found to occur with or shortly after SST increases. In addition, this is evident during the strongest El Niño years in DJF, and in-phase combinations of pIOD and El Niño events in JJA and SON. Such seasons subsequently dominated the annual scale signals of WP which suggests major interannual to decadal natural climate variability (i.e., ENSO, IOD, and PDO) acting in the Indo-Pacific are also dominant factors for inducing WP over the Indo-Pacific Ocean.

## Supplementary Information

Supplementary Information.
