# Supplementary material for: Positive relationship between seasonal Indo-Pacific Ocean wave power and SST
Source: Sci Rep. 2021 Aug 31;11:17419. doi: 10.1038/s41598-021-97047-3 (PMC8408234; doi:10.1038/s41598-021-97047-3)
Supplement: Supplementary file 1 — Supplementary Information. [file 41598_2021_97047_MOESM1_ESM.docx]

**Positive relationship between seasonal Indo- Pacific Ocean Wave Power and SST**

Sukhwinder Kaur^1^, Prashant Kumar^1^, Evan Weller^2^, Ian R. Young^3^

^1^Department of Applied Sciences, National Institute of Technology Delhi, Delhi, India

^2^School of Environment, University of Auckland, Auckland, New Zealand

^3^Department of Infrastructure Engineering, University of Melbourne, Australia

**Contents of this file**

**Nomenclature**

**Table S1**

**Figures S1 to S3**

| **Nomenclature** | |
| --- | --- |
| DJF | December, January, February |
| ENSO | El Niño–Southern Oscillation |
| G | Acceleration due to gravity |
| GEV | Generalized Extreme Value |
| IO | Indian Ocean |
| IOD | Indian Ocean Dipole |
| JJA | June, July, August |
| MAM | March, April, May |
| PDO | Pacific Decadal Oscillation |
| pIOD | positive Indian Ocean Dipole |
| PO | Pacific Ocean |
| PWP or Tp | Peak Wave Period |
| SO | Southern Ocean |
| SON | September, October, November |
| SST | Sea Surface Temperatures |
| SWH or Hs | Significant Wave height |
| ρ | Sea water mass density |
| Te | Energy period |
| WP | Wave Power |

**Table S1**: Seasonal contributions (%) in the SWH term (Hs^2^) and the PWP term (Tp) of Eq. (1) to WP of five hot spot regions over the 41-year period from 1979–2019.

| **Region** | **DJF** | | **MAM** | | **JJA** | | **SON** | |
| --- | --- | --- | --- | --- | --- | --- | --- | --- |
|  | Hs^2^ | Tp | Hs^2^ | Tp | Hs^2^ | Tp | Hs^2^ | Tp |
| **Arabian Sea**  (0°–30°N,20°E-78°E) | 21.49 | 78.51 | 18.09 | 81.90 | 45.88 | 54.12 | 22.30 | 77.69 |
| **Bay of Bengal**  (0°–30°N,78°E-105°E) | 17.88 | 82.12 | 20.80 | 79.19 | 33.78 | 66.22 | 25.21 | 74.79 |
| **Southern Indian Ocean**  (65°S–30°S, 20°E –120°E) | 69.57 | 30.43 | 75.25 | 24.75 | 78.86 | 21.14 | 75.11 | 24.89 |
| **North Pacific**  (25°N–65°N, 120°E–70°W) | 75.18 | 24.82 | 64.72 | 35.28 | 42.76 | 57.24 | 67.35 | 32.65 |
| **South Pacific**  (65°S–30°S, 120°E–110°W) | 66.72 | 33.28 | 73.95 | 26.05 | 75.49 | 24.51 | 72.13 | 27.87 |

**
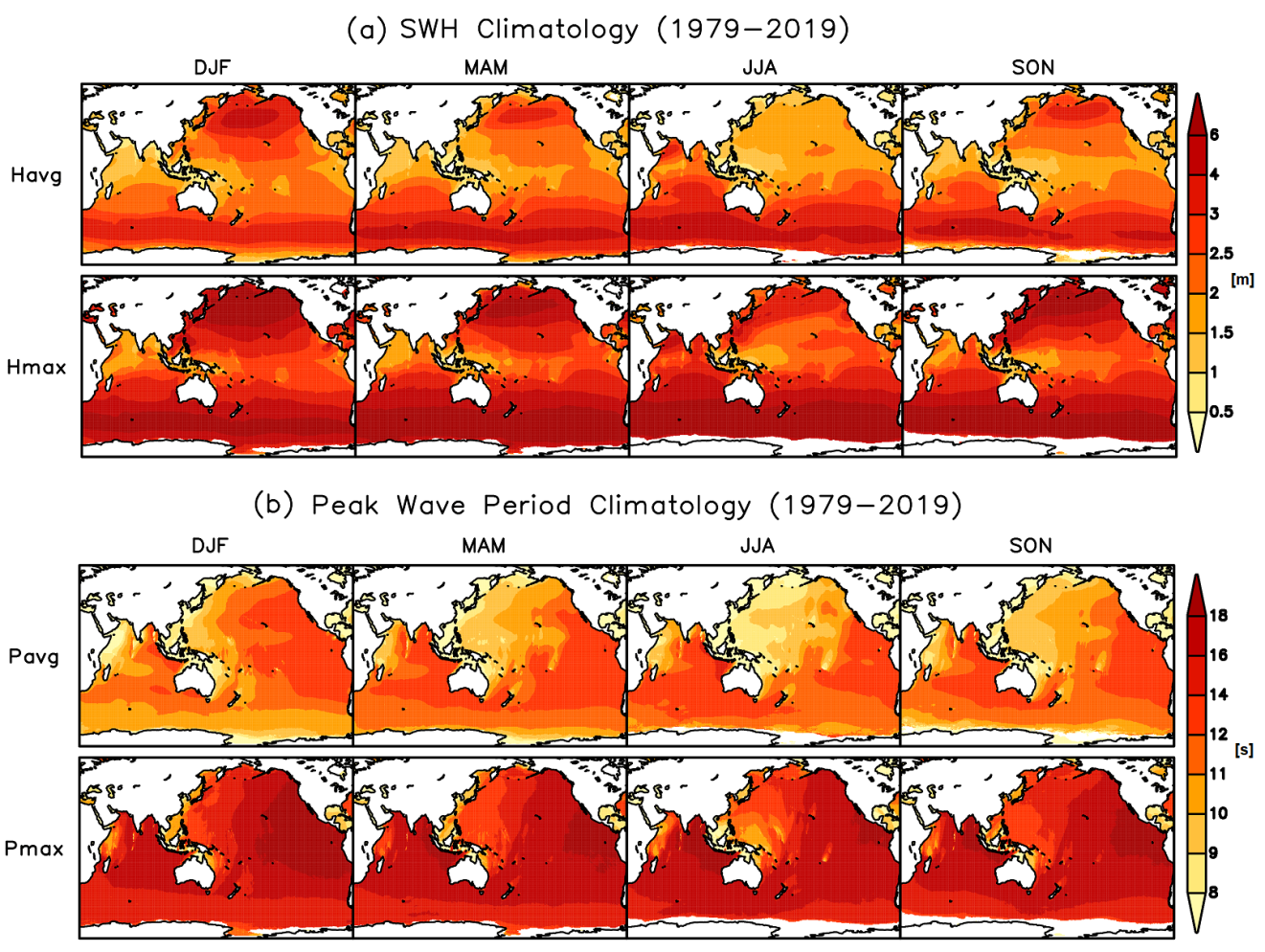
**

**Figure S1**. Climatology patterns of the seasonal mean and extreme (a) SWH and (b) PWP in the Indo-Pacific Ocean during 1979-2019. The units of SWH and PWP are given as meter (m) and seconds (s), respectively.


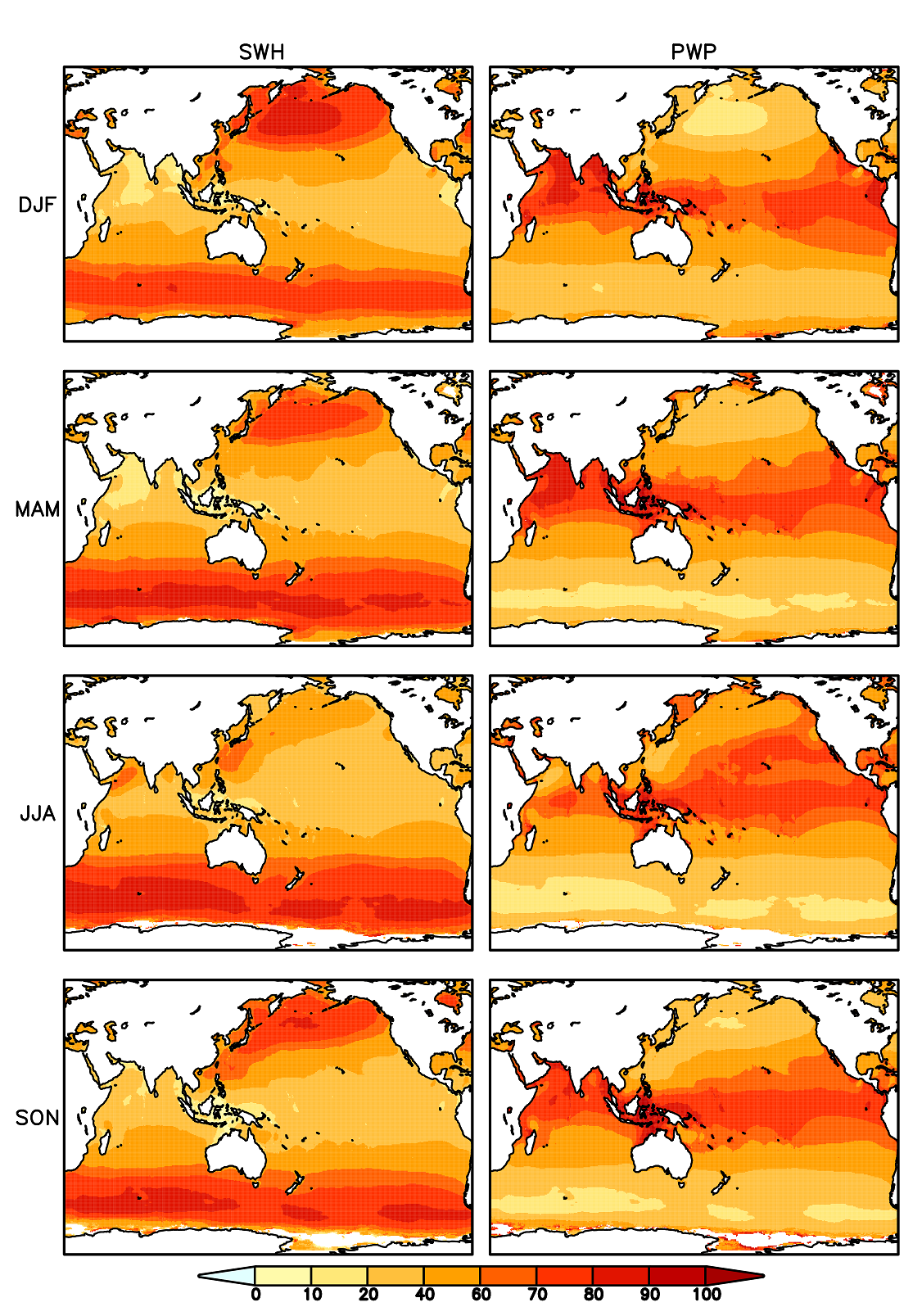


**Figure S2**. Spatial patterns of the seasonal contributions (%) of the SWH term (Hs^2^) and the PWP term (Tp) in Eq. (1) to WP over the Indo–Pacific Ocean during 1979–2019.


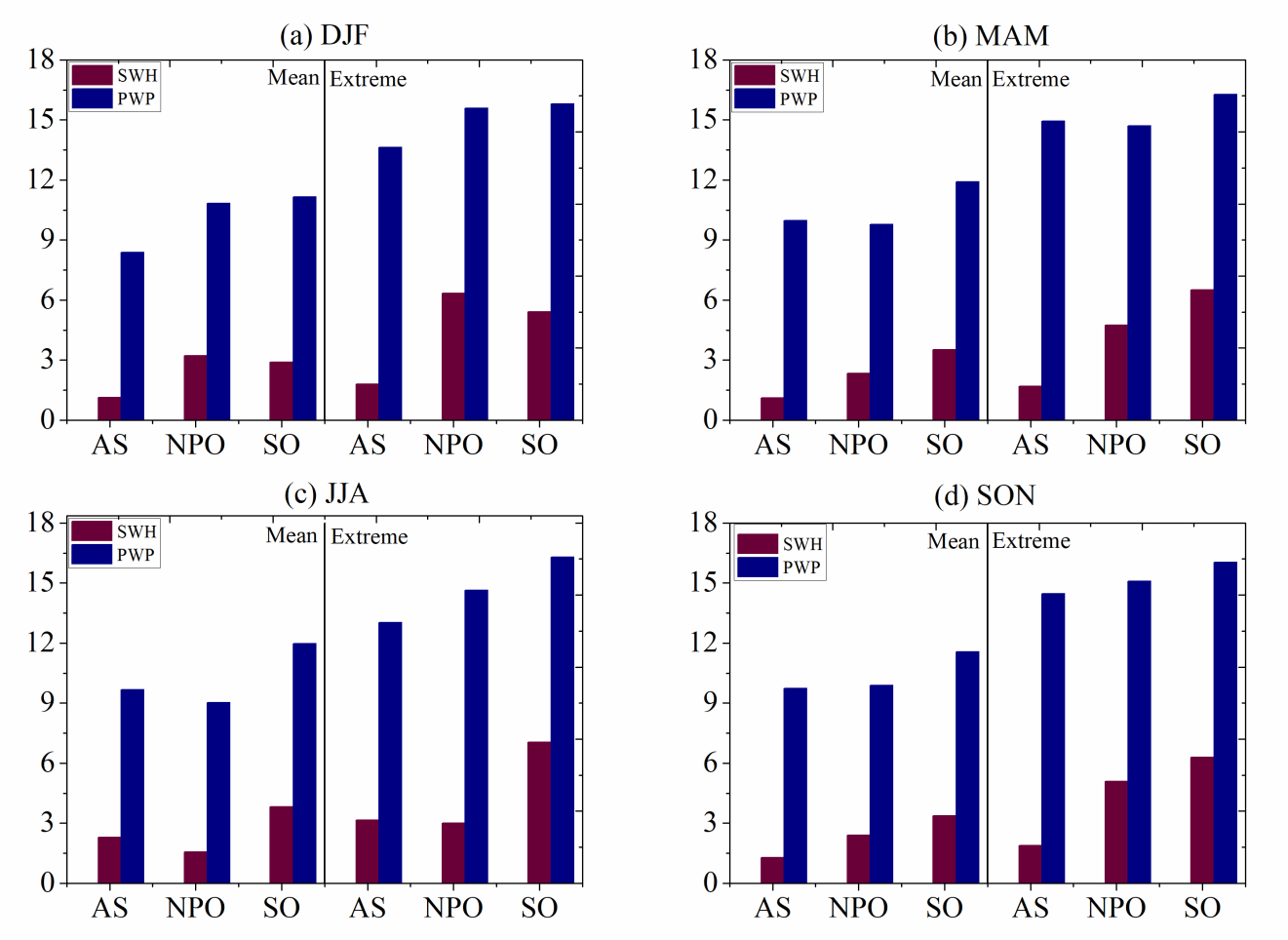
 **Figure S3.** The seasonal mean and extreme SWH and PWP for (a) DJF, (b) MAM, (c) JJA, and (d) SON over the Indo-Pacific Ocean during 1979–2019. The units of SWH and PWP are given as meter (m) and seconds (s), respectively.
